# Perceived Stigma and Quality of Life in Binary and Nonbinary/Queer Transgender Individuals in Italy: The Mediating Roles of Patient–Provider Relationship Quality and Barriers to Care

**DOI:** 10.3390/ejihpe15060113

**Published:** 2025-06-17

**Authors:** Selene Mezzalira, Gianluca Cruciani, Maria Quintigliano, Vincenzo Bochicchio, Nicola Carone, Cristiano Scandurra

**Affiliations:** 1 Department of Humanities, University of Naples Federico II, 80138 Naples, Italy; selene.mezzalira@unina.it (S.M.); cristiano.scandurra@unina.it (C.S.); 2 Department of Systems Medicine, University of Rome Tor Vergata, 00133 Rome, Italy; gianluca.cruciani@uniroma2.it (G.C.); maria.quintigliano@uniroma2.it (M.Q.); 3 Department of Humanities, University of Calabria, 87036 Rende, Italy; vincenzo.bochicchio@unical.it

**Keywords:** transgender, nonbinary, queer, perceived stigma, patient–provider relationship quality, barriers to care, quality of life

## Abstract

Among transgender binary and nonbinary/queer (TNBQ) individuals, perceived stigma has been documented to be significantly associated with health disparities that diminish overall quality of life. The present study examined the serial mediating roles of patient–provider relationship quality and perceived barriers to care in the association between perceived stigma and quality of life among TNBQ individuals residing in Italy. Data were collected from 132 TNBQ participants aged 18–60 years (*M* = 28.52, *SD* = 8.57) through an online survey assessing perceived stigma, patient–provider relationship quality, perceived barriers to care, and quality of life. A serial mediation model was analyzed using Model 6 of the SPSS Macro Process, version 29, and separately applied to two subgroups of TNBQ participants (i.e., binary and nonbinary) to detect potential differences. Findings indicated that in both groups (i.e., binary and nonbinary populations), when considered independently, only perceived barriers to care—but not patient–provider relationship quality—mediated the relationship between perceived stigma and quality of life. A serial mediation effect was also observed, wherein the relationship between perceived stigma and quality of life was mediated sequentially through patient–provider relationship quality and barriers to care, but only for the binary group. These findings hold significant clinical relevance, as improving the perceived quality of patient–provider relationships may help reduce perceived barriers to healthcare access. In turn, this may attenuate the detrimental effects of perceived stigma on the quality of life among TNBQ individuals.

## 1. Introduction

The terms “transgender,” “nonbinary,” and “queer” (TNBQ) refer to individuals whose gender identities do not conform to cisnormative, binary expectations, which assume an alignment between the sex assigned at birth and the gender with which they identify (Beek et al., 2016). TNBQ populations are well-documented as being disproportionately affected by health disparities, both within broader societal structures (Fredriksen-Goldsen et al., 2014) and within healthcare systems (Frohard-Dourlent et al., 2017; Mezzalira et al., 2024, 2025). In healthcare settings in particular, TNBQ individuals are especially vulnerable to perceived stigma, which constitutes a form of chronic, socially grounded minority stress. This form of stress compounds the everyday challenges that TNBQ individuals face as members of marginalized communities (Frost & Meyer, 2023; I. H. Meyer, 2003; Testa et al., 2015).

TNBQ individuals experience distinct developmental trajectories with regard to both identity exploration and affirmation, as they must navigate societal cisnormativity and pervasive gender binary assumptions on a daily basis. In addition to cisnormative pressures, Johnson (2016) introduced the concept of *transnormativity*—a hegemonic framework in which TNBQ identities are evaluated according to binary norms that privilege certain transgender experiences (typically those that conform to binary gender roles and involve medical transition) while marginalizing those that deviate from these expectations. As a result, not only transgender individuals broadly, but especially nonbinary and queer individuals, are compelled to negotiate their identities within social contexts that demand conformity to transnormative standards. This negotiation often requires seeking out or creating social spaces that resist these normative constraints, thereby enabling the expression of diverse gender identities (Bradford & Syed, 2019). One of the most severe outcomes of these structural and societal challenges is the heightened vulnerability of TNBQ individuals to transphobic and transnegative hate crimes. In this regard, Italy ranks poorly among European countries, reflecting the significant risks and discrimination faced by TNBQ individuals in Italian society (ILGA, 2024).

Notably, adopting a transnormative framework may inadvertently suggest that TNBQ individuals constitute a homogenous group, thereby overlooking the considerable diversity that characterizes this population (Bradford & Catalpa, 2019). In reality, TNBQ individuals exhibit a wide range of personal and identity-related differences, particularly with respect to gender identity and lived experiences. Individuals situated within the nonbinary and queer spectrum often report distinct experiences compared to their binary transgender counterparts and are disproportionately affected by poorer health outcomes (Tatum et al., 2020). For example, nonbinary youth face greater barriers to healthcare access—including specialized gender-affirming services—and frequently report lower confidence in expressing their identities and communicating their health needs (Clark et al., 2018). In addition, the gender affirmation pathways of nonbinary individuals tend to be less linear and more flexible than those of binary transgender individuals. These pathways often begin later in life and lack a clearly defined endpoint, reflecting the diverse ways in which nonbinary people navigate gender affirmation (Matsuno, 2019; Fiani & Han, 2019). The relative invisibility of nonbinary identities further contributes to the perception that their gender expressions are less legitimate or valid compared to binary identities (Bradford & Syed, 2019). Consequently, TNBQ individuals demonstrate substantial variability in how they confront and challenge binary gender, conceptualize identity, and understand gender fluidity. This diversity underscores the importance of recognizing the multiplicity of gender experiences within the TNBQ population, rather than subsuming them under a singular, transnormative narrative (Catalpa et al., 2019).

Perceived stigma—defined as the experience and anticipation of being labeled, excluded, stereotyped, and discriminated against based on one’s identity within contexts of power imbalance (Link & Phelan, 2001)—is often perpetuated through dominant discourses rooted in heteronormative and cisnormative societal beliefs (Velasco, 2022). These discourses operate across multiple socio-ecological levels (Hatzenbuehler & Link, 2014), thereby reinforcing the systemic marginalization of TNBQ identities (White Hughto et al., 2015). Perceived stigma is a well-documented contributor to the health disparities experienced by marginalized populations, including TNBQ individuals (King et al., 2020; Scandurra et al., 2019). Although research has emphasized the considerable resilience that TNBQ individuals often mobilize—particularly within healthcare settings (Lacombe-Duncan et al., 2020)—perceived stigma continues to exert harmful effects on mental health and overall quality of life (Bockting et al., 2013). Quality of life, in this context, refers to individuals’ subjective evaluation of their life positions, relative to their sociocultural and environmental circumstances (Skevington et al., 2004). However, despite growing evidence on the impact of stigma, the specific mechanisms through which perceived stigma translates into diminished quality of life remain only partially understood within the existing scientific literature.

In healthcare contexts, perceived stigma is reinforced by the power imbalance characterizing the patient–provider relationship (Cruciani et al., 2024; Poteat et al., 2013). Stigma is associated with TNBQ individuals’ experienced barriers in healthcare access (Berrian et al., 2025) and lower levels of healthcare services utilization (Whitehead et al., 2016). In turn, these factors contribute to producing and maintaining the health inequalities which TNBQ individuals are subject to, leading to poorer outcomes in terms of health and quality of life (Velasco et al., 2022). Indeed, TNBQ individuals frequently mention the providers’ professionality (e.g., using the correct names and pronouns; Dolan et al., 2020), preparedness to meet TNBQ-specific health needs, and attitudes promoting privacy and confidentiality as factors improving a patient–provider relationship informed by cultural humility and competence (Reeves et al., 2024). In contrast, healthcare professionals’ lack of knowledge and preparedness, as well as attitudes leading to the pathologization of diverse gender identities, are negatively voiced as detrimental to the perceived quality of the patient–provider relationship (Kattari et al., 2019). In turn, negative experiences in healthcare encounters decrease TNBQ individuals’ health-seeking behaviors (Falck & Bränström, 2023) and are thus likely to increase healthcare barriers in terms of dimensions of accessibility, acceptability, contact, and availability of service (Berrian et al., 2025).

In addition to demonstrating how aspects of the patient–provider relationship—such as effective and affirming communication (Ruben et al., 2019)—can buffer the negative effects of perceived stigma and discrimination on health-related outcomes, research has also highlighted the mediating role of relationship quality in the link between individual antecedents (e.g., stigma and discrimination) and healthcare outcomes (Anderson et al., 2019; Owen et al., 2011; Roos & Werbart, 2013). This mediating function has been observed not only in the general population but also among individuals marginalized on the basis of diverse sexual identities (Carone et al., 2025).

Research has also indicated that sociodemographic variables like age and education influence healthcare-related variables such as patient engagement in health-seeking behaviors and satisfaction with the quality of healthcare encounters. For instance, older and more educated individuals tend to be more proactive in seeking healthcare support than their younger and less educated counterparts (Sun et al., 2019). However, as opposed to those with higher educational levels and those who access healthcare services more often, older individuals tend to have lower expectations of the quality of healthcare services (Manulik et al., 2018). Among TNBQ populations, healthcare-seeking behaviors have also been shown to vary as a function of age. This variation may be attributed to the fact that, compared to younger individuals, TNBQ adults and older individuals are more likely to have navigated key developmental milestones—such as the coming out process, gender affirmation, and body-related experiences—which can enhance their readiness to access and utilize healthcare services (Porter et al., 2016). As to the individual’s educational level, findings are mixed, with studies reporting higher engagement in healthcare in individuals with higher educational levels (Sun et al., 2019) and others reporting less positive experiences with healthcare services among less educated individuals (Afzal et al., 2014). However, research specifically focusing on TNBQ populations has shown that higher levels of avoidance and delay of healthcare access and utilization were enacted by older and less educated TNBQ individuals (Goldenberg et al., 2020).

Significant associations also exist between poorer self-reported health and more negative perceptions of barriers to care (Premji et al., 2018), including the lack of available resources in the individuals’ housing area (Renner et al., 2021), and between the presence of a medical condition and a higher likelihood of reporting unmet health needs (Zhang et al., 2018). Finally, having a habitual source of primary care, defined as a “medical home” (Starfield & Shi, 2004) such as the doctor’s office or a community clinic, has been argued to foster good health and management of routine as well as chronic diseases, thus representing an important indicator of healthcare access and utilization (Hoffman & Paradise, 2008). Therefore, in our study, we aimed to further verify the potential role of sociodemographic (i.e., age, educational level) and health-related variables (i.e., presence of chronic diseases, habitual source of primary care) in the association between perceived stigma and quality of life through the perceived patient–provider relationship quality and experienced barriers to care.

Research is needed to clarify the multiple mechanisms through which TNBQ individuals’ perceived stigma impacts their quality of life, especially as it concerns healthcare-related variables such as the perceived quality of the patient–provider relationship and the experience of barriers to care. Therefore, investigating the perceived quality of healthcare encounters and experienced barriers to access and utilization of healthcare services might add further insight into how perceived stigma affects TNBQ individuals’ quality of life. Indeed, it is plausible that a high-quality patient–provider relationship might help TNBQ individuals experience fewer barriers to care, aiding them in more effectively meeting their health needs and, therefore, improving their quality of life.

Contextualizing this type of research is essential because TNBQ individuals experience healthcare services in very different ways, depending on the social, cultural, and political contexts where they live. These environments shape not only access to care but also the quality of interactions with healthcare providers, the availability of gender-affirming services, and the levels of perceived stigma. Along this line, the current research has been conducted in Italy, a country where the lack of governmental laws protecting LGBT+ populations and especially TNBQ individuals adds to the homo- and transnegative assumptions governing societal structures and systems (Feo, 2022). Indeed, in 2024, Italy was ranked in 35th place in Europe as to the rights and freedom of LGBT+ individuals within society (ILGA, 2024). Accordingly, research has shown that Italy is still a highly cis- and heteronormative country, where individuals with minoritized sexual and especially gender identities face pervasive societal homo- and transphobic attitudes (Anzani et al., 2024; Bochicchio et al., 2024; Bourelly et al., 2024; Marconi et al., 2025; Prunas et al., 2018; Scandurra et al., 2018).

In the context of the Italian healthcare system, the National Health Service (Servizio Sanitario Nazionale, SSN) provides universal coverage and, in principle, ensures equal treatment for all citizens and legal foreign residents. Access to healthcare services is primarily free of charge, with the exception of copayments required for certain specialized procedures (Ferré et al., 2014). Nonetheless, despite the system’s foundational commitment to equitable healthcare access, substantial disparities persist across population groups (Costa et al., 2003). Significant inequalities continue to affect access to specialist and inpatient care, often reflecting regional, gender-based, and socioeconomic differences (Tikkanen et al., 2020). With respect to TNBQ individuals, the Italian healthcare system is frequently characterized as inadequately equipped to meet their specific needs. This inadequacy is largely attributed to healthcare providers’ limited cultural competence and the absence of systematic training programs focused on TNBQ health issues (Marconi et al., 2024). Moreover, gender-affirming procedures in Italy are regulated by Law No. 164/1982—amended in 2011 and 2014—which requires individuals to obtain judicial authorization in order to change their legal name on official documents. However, prolonged waiting periods and associated financial costs constitute significant barriers to timely legal recognition, often exacerbating fears of discrimination, particularly in healthcare settings (Bassetti, 2019). Furthermore, the Italian legal framework lacks nationally standardized guidelines for gender-affirming medical care. Instead, such practices typically emerge from the independent efforts of local healthcare units, resulting in inconsistent and fragmented service provision across the country.

Research on the healthcare experiences of TNBQ individuals in Italy has consistently documented a high prevalence of discrimination within healthcare settings. For example, Marconi et al. (2024) reported that over one-third of TNBQ participants (*n* = 959) experienced discrimination in healthcare contexts, with the most frequently cited issues being healthcare providers’ lack of knowledge regarding transgender health and the use of inappropriate terminology. Notably, among nonbinary participants, individuals assigned female at birth (AFAB) reported higher rates of discrimination compared to those assigned male at birth (AMAB). Discrimination rooted in providers’ lack of preparedness is further supported by findings from Leone et al. (2023), in which only 19% of oncology healthcare providers (*n* = 305) felt competent in transgender care. These providers often lacked awareness of the health disparities affecting TNBQ populations and the structural barriers they face in accessing care. This was echoed by patient respondents (*n* = 190), who identified providers’ limited experience and knowledge as key contributors to discriminatory encounters. Similarly, Della Pelle et al. (2018) found that among a large sample of nurses (*n* = 824), female nurses expressed more positive attitudes toward sexual and gender minorities, although male nurses reported greater confidence in their preparedness to care for LGBT individuals. Collectively, these findings underscore a widespread lack of competence and the persistence of discriminatory, authoritarian, and paternalistic behaviors among healthcare professionals in Italy (Costa & Rotundo, 2024). Against this backdrop, examining healthcare-related variables—such as the perceived quality of patient–provider relationships and experienced barriers to care—may be crucial for informing policies and practices aimed at reducing stigma and discrimination toward TNBQ individuals within Italian healthcare systems.

**Figure 1:**
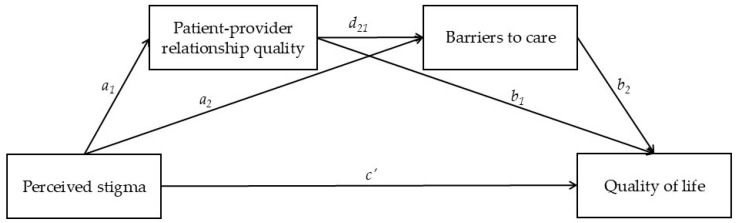
Hypothesized serial mediation model.

Building on this foundation, the present study aimed to contribute to a deeper understanding of the mechanisms through which stigma impacts TNBQ individuals’ quality of life. Specifically, it investigated the potential mediating roles of perceived patient–provider relationship quality and experienced barriers to care in the association between perceived stigma and quality of life among a sample of TNBQ individuals residing in Italy. Given the premises outlined above, which highlight the substantial differences between binary and nonbinary individuals in terms of quality of life and healthcare experiences, we decided to perform our analyses independently for each group to detect potential differences in the associations among variables. For both gender identity categories, we first hypothesized that perceived stigma would be negatively associated with quality of life (Hypothesis 1; *c’*). We then hypothesized that both the patient–provider relationship quality (Hypothesis 2; a_1_*b_1_) and barriers to care (Hypothesis 3; a_2_*b_2_) would mediate the relation between perceived stigma and quality of life. Finally, we hypothesized that the perceived quality of the patient–provider relationship and experienced barriers to care would play a serial mediating role in the relation between perceived stigma and quality of life (Hypothesis 4; a_1_*d_21_*b_2_). Specifically, we hypothesized that higher levels of perceived stigma would be associated with lower perceived quality of the patient–provider relationship, which, in turn, would be positively associated with the perception of greater barriers to care. This sequential pathway was expected to ultimately exert a negative impact on quality of life. The hypothesized model is depicted in Figure 1.

## 2. Materials and Methods

### 2.1. Procedures and Participants

Data were collected via a web-based survey hosted on Qualtrics, which was distributed through social media platforms (e.g., Facebook) and by reaching out to associations representing the TNBQ community. In addition, the implementation of a snowball sampling procedure aimed at encouraging all potentially interested participants to share the survey with the people belonging to their networks. Four inclusion criteria were applied: (1) self-identifying as a person within the TNBQ spectrum of identities; (2) being ≥18 years old (i.e., the Italian age for consent); (3) living in Italy; and (4) having had access to at least one healthcare service in Italy within the last 5 years. By clicking on the link provided, participants could read the informed consent, the study aims and objectives, the researchers’ information, and the benefits and risks of the study. Participants were informed that the survey would be anonymous and that they had the right to withdraw from it at any time if they wished to.

The collected data were processed by ensuring the anonymity of the participants with every precaution necessary to avoid their identification. The data were stored by the principal investigator (i.e., Nicola Carone) using secure technological instruments (e.g., employing encrypted passwords) in accordance with the principles of Article 5 EU Regulation 2016/679. The study received ethical approval from the Ethical Committee of the University of Naples “Federico II” (protocol no. 10/2024) and the Territorial Ethics Committee Lazio Area 2 (protocol no. 197.24 CET2 utv). The study adhered to the EU General Data Protection Regulation and the ethical principles outlined in the Declaration of Helsinki for medical research involving human subjects. Privacy and data security were ensured in compliance with Italian law 196/2003.

### 2.2. Measures

#### 2.2.1. Sociodemographic and Health-Related Characteristics

The sociodemographic characteristics assessed in the current study included age, sex assigned at birth, gender identity (trans woman, trans man, nonbinary, and queer person), educational level (1 = ≤high school; 2 = ≥college), and ethnicity (1 = Caucasian; 2 = non-Caucasian). Given the low number of participants identifying as queer, the latter were included in the nonbinary group. Two additional questions evaluated health-related aspects. First, the presence of chronic diseases was assessed through a dichotomous question: if participants responded “yes,” they could also report which chronic disease(s) they suffered from. Second, a two-step process (Green et al., 2022) was employed to determine if participants had a habitual source of primary care. Survey questions included the following: “Is there a place that you usually go to when you are sick or need advice about your health?” To this question, participants responded “no” or “yes.” If participants responded “yes,” a question followed, asking about the source of care: “What kind of place is it (clinic, doctor’s office, hospital out-patient department, hospital emergency room or other)?” Those who reported “hospital emergency room” as their habitual source of care were categorized as not having a habitual source of care (Pitts et al., 2010), whereas those who reported “other” as their habitual source of care were treated as missing data, as it was not possible to determine whether “other” was a primary care service. All other response options were treated as having a primary care service as a habitual source of primary care.

#### 2.2.2. Perceived Stigma

Perceived stigma was assessed through the *Perceived Stigma Scale* (PSS), a 6-item questionnaire based on the original measure developed by Link (1987) and later adapted by I. H. Meyer et al. (2008). Participants were asked the following: “The following statements refer to a person like you; by ‘person like you’ we mean a person with the same gender identity as you. We would like you to respond based on how you feel that people (in general) would think in terms of this category.” An example item is “Most people have a low opinion of a person like me,” to which participants responded on a 4-point Likert scale ranging from “strongly disagree” to “strongly agree.” Cronbach’s alpha was 0.87.

#### 2.2.3. Patient–Provider Relationship Quality and Barriers to Care

The perceived quality of the patient–provider relationship and the perception of barriers to care were measured using two distinct subscales of the *Multidimensional Measure of Health Care Access LGBTQ* (MMHCA; Romanelli et al., 2024), comprised of 19 items to which participants could respond on a 4-point Likert scale from “never” to “always.” Example items are, respectively, “I would feel comfortable talking about personal problems with a professional” and “I didn’t trust and believe in doctors.” Cronbach’s alpha was 0.75 for the subscale assessing the patient–provider relationship quality and 0.76 for the subscale addressing barriers to care.

#### 2.2.4. Quality of Life

Quality of life was measured using the EQ-5D-5L (EuroQol Research Foundation, 2019), a 5-item questionnaire of various quality of life-related dimensions including mobility, self-care, activity, pain, and anxiety, to which participants indicated on a 5-point Likert scale the extent to which they encountered the corresponding limitations, selecting from “no difficulties at all” to “extreme difficulty.” Cronbach’s alpha was 0.71.

### 2.3. Statistical Analyses

Statistical analyses were performed using the Statistical Package for the Social Sciences (SPSS) version 29.0. Preliminary descriptive statistics of participants’ sociodemographic and health-related characteristics (means and standard deviations) were examined. Bivariate correlations between the study variables (i.e., sociodemographic and health-related information, perceived stigma, patient–provider relationship quality, barriers to care, and quality of life) were then calculated using Pearson’s correlation coefficient. Correlations were considered significant if *p* < 0.05.

Serial mediation analyses were performed using the Macro PROCESS (Model 6) for SPSS (Hayes, 2018). The independent variable was perceived stigma, the mediators were the quality of the patient–provider relationship and barriers to care, and the dependent variable was quality of life. The strength of the indirect, direct, and total effects was estimated, and significance was tested by producing 95% confidence intervals (CIs). Effects were considered significant if the upper and lower boundaries of the 95% CIs did not contain zero. Age, educational level, presence of chronic diseases, and having a habitual source of primary care were inserted as covariates, but not ethnicity, given that the vast majority of participants (97%, *n* = 128) were Caucasian.

Finally, a post hoc Monte Carlo power simulation was computed to obtain the statistical power of the results for the indirect effects using the shiny and MASS add-on R packages (Schoemann et al., 2017). Notably, as previously mentioned, the analyses were conducted on two groups representing binary and nonbinary identities. Due to the limited number of participants who self-identified as queer (*n* = 19), individuals identifying as nonbinary and those identifying as queer were combined into a single nonbinary group for the purposes of statistical analysis.

## 3. Results

### 3.1. Participants’ Characteristics

The total sample consisted of 132 TNBQ participants aged 18–60 years (*M* = 28.5; *SD* = 8.6). Binary individuals’ age range was 19–60 (*M* = 31; *SD* = 11.1), whereas in nonbinary people, the age range was 18–47 (*M* = 27.1; *SD* = 6.4). Regarding the participants’ gender identity characteristics, 36.4% (*n* = 48) were transgender binary individuals, whereas 63.6% (*n* = 84) were nonbinary people. Most participants (58.3%, *n* = 77) were highly educated, holding at least a university degree. Furthermore, 27.3% of participants (*n* = 36) were affected by at least one chronic disease; specifically, the most common disease reported among these individuals (*n* = 10, 27.7%) was rheumatoid arthritis. Finally, 81.1% (*n* = 107) of participants reported having a habitual source of primary care. The details of the participants’ sociodemographic and health-related characteristics are outlined in Table 1.

### 3.2. Descriptive Statistics and Bivariate Correlations

The results showed that in the total sample, perceived stigma correlated negatively with the quality of the patient–provider relationship and quality of life and positively with the perception of barriers to care. Furthermore, the patient–provider relationship quality was correlated negatively with barriers to care and positively with quality of life. Finally, experienced barriers to care were correlated negatively with quality of life. Table 2 presents the correlations among the variables under investigation, as well as the results of independent samples *t*-tests comparing binary and nonbinary groups. The findings indicate that nonbinary individuals report significantly lower levels of quality of life and perceive the patient–provider relationship as less positive compared to their binary counterparts.

### 3.3. Direct and Indirect Associations Between Perceived Stigma and Quality of Life Through Patient–Provider Relationship Quality and Barriers to Care

**Figure 2:**
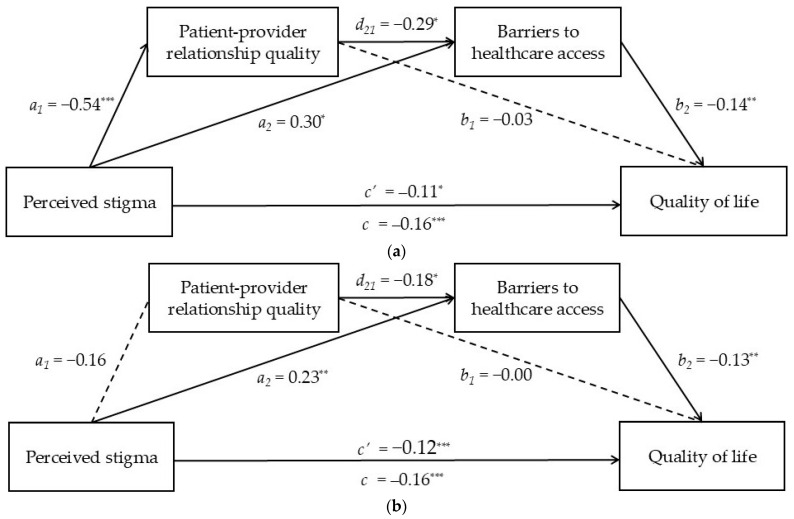
(**a**) Results of the serial mediation analysis in the binary population (*n* = 48). (*Notes:* * = *p* < 0.05; ** = *p* < 0.01; *** = *p* < 0.001). (**b**) Results of the serial mediation analysis in the nonbinary population (*n* = 84). (*Notes:* * = *p* < 0.05; ** = *p* < 0.01; *** = *p* < 0.001).

The serial mediation analysis was performed to investigate whether the association between perceived stigma and quality of life was mediated by the perceived patient–provider relationship quality and barriers to care, after controlling for all covariates (i.e., age, educational level, presence of diseases, and habitual source of primary care). The serial mediation model was tested for each TNBQ subpopulation (i.e., binary and nonbinary individuals). The effects of the paths linking perceived stigma to each mediator and quality of life are shown in Figure 2a,b for the binary and nonbinary populations, respectively.

For both the binary and the nonbinary populations, perceived stigma had a negative impact on quality of life (Hypothesis 1). However, taken separately, only the perception of barriers to care (Hypothesis 3), but not the perceived quality of the patient–provider relationship (Hypothesis 2), mediated the association between perceived stigma and quality of life. In turn, the third indirect path (perceived stigma → patient–provider relationship quality → barriers to care → quality of life), which represents the main hypothesis of the current study (Hypothesis 4), was significant only for the binary group, suggesting that only for these individuals, higher levels of perceived stigma decrease quality of life by decreasing the perceived patient–provider relationship quality, which in turn decreases the perception of barriers to care (i.e., the hypothesized mediators).

As to the control variables, for binary people, quality of life was impacted positively by age (*b* = 0.01, *p* = 0.025, *95% CIs* = 0.00, 0.01) and negatively by the presence of chronic diseases (*b* = −0.16, *p* = 0.017, *95% CIs* = −0.30, −0.03) and habitual primary care access (*b* = −0.15, *p* = 0.022, *95% CIs* = −0.27, −0.02). Instead, for nonbinary people, educational level had a positive impact on quality of life (*b* = 0.08, *p* = 0.005, *95% CIs* = 0.03, 0.14).

In the binary group, Monte Carlo power analysis for indirect effects showed a small power of 5%, 6%, and 6% for the paths a_1_*b_1_, a_2_*b_2_, and a*d*b_2_, respectively (based on 95% CI and 2000 bootstrap replications; Schoemann et al., 2017). Likewise, in the nonbinary group, Monte Carlo power analysis for indirect effects showed a small power of 1%, 11%, and 2% for the paths a_1_*b_1_, a_2_*b_2_, and a*d*b_2_, respectively. Table 3 reports regression coefficients, standard errors, 95% CIs, and *p*-values for all paths in the two subpopulations (i.e., binary and nonbinary individuals and the total sample).

## 4. Discussion

The present study examined the mediating roles of perceived patient–provider relationship quality and experienced barriers to care in the association between perceived stigma and quality of life among a sample of TNBQ individuals residing in Italy. Given the distinct experiences of binary and nonbinary individuals, analyses were conducted separately for each group. Preliminary independent samples *t*-tests revealed significant differences between the two groups. Specifically, nonbinary individuals reported a less positive perception of the patient–provider relationship compared to their binary counterparts. This result is consistent with prior research showing that nonbinary individuals are more frequently subjected to misgendering by healthcare professionals than binary individuals (Goldberg et al., 2019).

In addition, nonbinary participants reported a lower overall quality of life relative to binary individuals. This result is consistent with existing literature suggesting that nonbinary individuals often face greater challenges in expressing their identities due to prevailing norms of prejudicial transnormativity (Johnson, 2016). These challenges are rooted in the dominance of binary gender assumptions within society, which complicate the identity negotiation process for nonbinary individuals—particularly when compounded by intersecting systems of oppression that restrict the free expression of diverse gender identities (Bradford & Syed, 2019). As a result, nonbinary individuals tend to experience lower levels of societal acceptance and greater invisibility within institutional and cultural structures (Bradford & Syed, 2019). In line with these findings, previous research has documented that nonbinary individuals face increased barriers to accessing healthcare (Clark et al., 2018) and report poorer health outcomes compared to binary individuals (Tatum et al., 2020).

Supporting our first hypothesis, perceived stigma was found to have a significant negative impact on quality of life in both groups (i.e., binary and nonbinary), consistent with previous research identifying stigma as a fundamental social determinant of health that adversely affects TNBQ individuals not only within broader societal contexts but particularly within healthcare settings (Cesconetto Coswosck et al., 2023). Beyond its direct association with quality of life, perceived stigma was found to negatively influence the perceived quality of the patient–provider relationship for binary individuals only and the experience of barriers to care for both groups. Prior research has emphasized that the quality of patient–provider interactions for transgender and gender diverse individuals may be compromised by perceived power imbalances, cisnormative assumptions, and the pathologization of gender diversity (H. M. Meyer et al., 2020). These dynamics can erode trust and foster a sense of marginalization within clinical encounters. In contrast, affirming and culturally competent relationships with healthcare providers have been shown to create safer environments in which TNBQ patients feel more comfortable disclosing their health concerns, reducing the perception of providers as gatekeepers. Furthermore, perceived stigma is closely linked to adverse health outcomes that arise from reduced access to and utilization of healthcare services (Clark et al., 2018; Goldenberg et al., 2020). In this context, stigma contributes to a cycle of avoidance and disengagement from care, often rooted in anticipatory fear of discrimination and rejection (Ritterbusch et al., 2018). These findings underscore the importance of addressing stigma at both interpersonal and structural levels to improve healthcare experiences and quality of life outcomes for TNBQ individuals.

Our findings indicated that when examined independently, only experienced barriers to care (Hypothesis 3)—and not the perceived quality of the patient–provider relationship (Hypothesis 2)—mediated the association between perceived stigma and quality of life in both groups. These results suggest that in isolation, the perceived quality of the patient–provider relationship does not play as critical a role as barriers to care in explaining how perceived stigma impacts the quality of life among TNBQ individuals. This highlights the unique and substantial explanatory power of experienced barriers to care, even in the absence of other healthcare-related variables. One possible interpretation is that TNBQ individuals place considerable importance on the perceived availability of resources that support effective access to and utilization of healthcare services. These structural and systemic resources may be viewed as more essential for meeting health-related needs and achieving a satisfactory quality of life. As further discussed below, it is only when both perceived patient–provider relationship quality and experienced barriers to care are considered simultaneously that they offer meaningful insight into the mechanisms by which perceived stigma influences quality of life—though this joint mediating effect emerged solely in the binary group and not among nonbinary individuals (Hypothesis 4).

In this regard, the results of the present study confirmed our main hypothesis, indicating that the perceived patient–provider relationship quality and experienced barriers to care serially mediated the association between perceived stigma and quality of life, albeit only for the binary group. In fact, as opposed to the binary group (Figure 2a), the path linking perceived stigma and the patient–provider relationship quality was not significant in the nonbinary group (Figure 2b). This difference might be interpreted considering that, for nonbinary individuals, barriers to care may be more salient than the perceived quality of the patient–provider relationship. In other words, the relational space with the healthcare provider might be less significant given the substantial barriers to care that nonbinary people experience when accessing health services. This also means that the strength of the association between perceived stigma and quality of life in nonbinary individuals leaves room only for barriers to care as an explanatory variable, given that the quality of the patient–provider relationship does not mediate such association. Nonetheless, this finding should be better explored in future studies, which might more deeply investigate the role played by the patient–provider relationship quality in explaining the impact of perceived stigma on quality of life in nonbinary populations.

Among the covariates included in the analysis, several factors were found to significantly influence quality of life, with differences observed between the binary and nonbinary groups. For binary individuals, quality of life was positively associated with younger age, suggesting that younger individuals in this group reported higher quality of life. In contrast, the presence of chronic health conditions was negatively associated with quality of life, as might be expected given the general burden of chronic illness. Interestingly, habitual access to a primary care provider also showed a negative association with quality of life. One possible interpretation of this finding is that regular engagement with primary care may result in prolonged exposure to stigmatizing or discriminatory healthcare environments. This repeated exposure may diminish individuals’ perceived satisfaction with the care received and, consequently, their overall quality of life. In the nonbinary group, educational attainment emerged as a positive predictor of quality of life. This may reflect the role of higher education in enhancing health literacy and empowering individuals to more effectively articulate and advocate for their healthcare needs. Previous research has shown that individuals with higher educational levels are often treated with greater respect by healthcare providers (Kattari et al., 2019), which may contribute to a greater sense of validation and satisfaction with healthcare experiences, ultimately supporting better quality of life.

Overall, especially for the binary population, these findings indicate that although these individuals often demonstrate considerable resilience in the face of stigma—through strategies such as self-agency, self-advocacy, and strategic self-disclosure (Velasco et al., 2022)—perceived stigma nonetheless has the potential to diminish the perceived quality of the patient–provider relationship and reduce engagement in help-seeking behaviors (Lacombe-Duncan et al., 2020). This, in turn, may impede the fulfillment of health needs among TNBQ individuals (James et al., 2016), ultimately leading to poorer health outcomes and a reduced quality of life (Velasco et al., 2022). Accordingly, fostering more positive perceptions of the patient–provider relationship may help reduce perceived barriers to care, thereby enhancing the capacity of these individuals to mitigate the negative effects of stigma on their quality of life.

On this basis, the present findings underscore the significant, combined role of perceived patient–provider relationship quality and experienced barriers to care in explaining the impact of perceived stigma on the quality of life among trans binary individuals. The intertwining of these healthcare-related experiences highlights the complexity of the pathways through which stigma exerts its influence. Specifically, the quality of interactions with healthcare providers and the degree of perceived access-related barriers jointly shape the extent to which stigma affects well-being, emphasizing the multifaceted nature of healthcare experiences. This complex interplay suggests that stigma negatively influences quality of life by both undermining trust and rapport within the patient–provider relationship and intensifying structural and interpersonal barriers to care (von Vogelsang et al., 2016).

These findings add to previous research conducted in other contexts, which mainly focused on single dimensions related to the variables investigated in the current study. For instance, Zeluf et al. (2016) found that negative healthcare experiences were associated with lower quality of life among trans people in Sweden. Similarly, Boyer et al. (2022) showed that non-affirming healthcare experiences were associated with healthcare avoidance among TNBQ people, which likely influenced their health and well-being. Research conducted in the U.S. has indicated that the patient–provider communication quality mediated the association between perceived medical heterosexism and health-seeking behaviors in a sample of sexual minority women and nonbinary individuals (Tabaac et al., 2019). In addition, U.S. research has documented that when compared with binary people, queer and nonbinary individuals were less likely to have a doctor aware of their gender identity (Kattari et al., 2019), which was likely to influence the quality of the patient–provider relationship. Finally, in a study conducted in Brazil, stigma has been found to impact the patient–provider communication quality through oppression-related variables in a sample of trans women living with HIV (Amarante et al., 2024). However, to our knowledge, no prior study has specifically examined the serial mediating roles of perceived patient–provider relationship quality and experienced barriers to care in the association between perceived stigma and quality of life among transgender binary and nonbinary populations. As such, the present research offers a valuable contribution by shedding light on the potential pathways through which perceived stigma adversely impacts quality of life in these groups. This is particularly relevant within the Italian healthcare context, where differences between binary and nonbinary individuals in accessing and navigating healthcare services remain understudied.

In this vein, clinical and social policies must critically engage with TNBQ individuals’ perceptions of healthcare interactions, aiming to enhance the quality of the patient–provider relationship and reduce perceived barriers to accessing care. These efforts are essential for ensuring adequate satisfaction of health needs and, in turn, promoting improved quality of life outcomes. Notably, despite the detrimental effects of stigma, TNBQ individuals frequently mobilize considerable resilience and coping strategies—including the use of social support, self-advocacy, persistence in seeking care, and strategic identity disclosure—to navigate and resist stigmatizing environments (Seelman & Poteat, 2020). In this context, fostering more inclusive, affirming, and informed healthcare services may not only mitigate the impact of stigma but also reinforce and support these existing resilience capacities. In summary, the present study contributes to the existing literature by elucidating the critical interconnection of specific healthcare-related mechanisms—namely, perceived patient–provider relationship quality and experienced barriers to care—through which perceived stigma exerts a substantial influence on the quality of life among TNBQ individuals.

### 4.1. Limitations

The findings of the present study should be interpreted in light of several limitations. First, the cross-sectional design employed in the serial mediation analysis precludes the ability to draw causal inferences regarding the observed associations among variables. Future research employing longitudinal designs is necessary to establish the directionality and temporal sequencing of these relationships. Second, the relatively small sample size limits the generalizability of the findings and may have contributed to the small size of the observed indirect effects. This constraint reduces the statistical power to detect more robust mediation pathways and may hinder the identification of subtler group differences. As such, future studies with larger samples are warranted to more thoroughly investigate this pathway. Nevertheless, this study sought to be inclusive by incorporating trans binary and nonbinary individuals, thereby addressing the diversity of gender identities within the TNBQ population. This represents an important step beyond prior research that has predominantly focused on the experiences of binary individuals (Bower-Brown et al., 2023; Frohard-Dourlent et al., 2017).

One further limitation concerns the imbalance between the number of binary and nonbinary individuals, which future studies should consider to verify the significance of the associations between the variables investigated in groups more balanced in terms of numerosity. As another limitation, we should mention the demographic composition of the sample, which consisted primarily of Caucasian participants. This lack of ethnic diversity underscores the need for future research to better represent individuals from a range of ethnic backgrounds and to attend to the intersectional nature of identities that shape healthcare experiences. Additionally, future studies should aim to include more heterogeneous samples across various sociodemographic dimensions, including comparative analyses between TNBQ individuals and their cisgender counterparts, to more fully understand the unique and shared healthcare challenges across gender identities. A final limitation concerns the circumscribed context of our study (i.e., Italy), which is governed by rules quite different from other Western countries (e.g., the U.S.). Therefore, future studies might consider using cross-national surveys to compare different healthcare systems, thus highlighting more differentiated context-related differences.

### 4.2. Clinical Implications and Recommendations for Future Research

The present study offers a meaningful contribution to the literature on the mechanisms linking perceived stigma to quality of life among TNBQ populations. Specifically, it highlights the critical role of perceived patient–provider relationship quality and experienced barriers to care as mediating factors in this association, even though only for the binary group. These findings carry important clinical implications. Given that perceived stigma adversely affects TNBQ individuals not only within broader societal contexts but also—perhaps more acutely—within healthcare settings, addressing the quality of healthcare encounters becomes paramount. A more positive patient–provider relationship can reduce perceived barriers to care, thereby promoting improved access, better health outcomes, and, ultimately, enhanced quality of life.

Importantly, TNBQ individuals consistently report the need for improved access to care and express a desire to engage with healthcare providers who are less biased, less judgmental, and better informed about their specific health needs (Teti et al., 2021). In response to these needs, it is essential that targeted training programs be implemented to increase healthcare professionals’ competence in delivering inclusive and affirming care to TNBQ populations (Cruciani et al., 2024; Santamaria et al., 2024). Such training has the potential to reduce experiences of stigma and discrimination, enhance the accessibility and usability of healthcare services, and contribute to the more effective fulfillment of TNBQ individuals’ health needs, thereby improving their quality of life.

Ultimately, the findings of this study underscore the necessity of providing well-informed, compassionate, and culturally competent care to TNBQ individuals. A patient–provider relationship grounded in principles of confidentiality, autonomy, advocacy, and respect (Sundus et al., 2025) can help mitigate the impact of perceived stigma by reducing experienced barriers to care (Vermeir et al., 2018). Such care not only supports individual health outcomes but also contributes to the development of more inclusive healthcare systems that actively work to dismantle stigma and promote equity for marginalized communities such as TNBQ populations.

## 5. Conclusions

Understanding how stigma-related health disparities influence the quality of life of TNBQ individuals is essential for informing future clinical interventions aimed at promoting positive health outcomes within this population. The present study underscored the serial mediating roles of perceived patient–provider relationship quality and experienced barriers to care in the association between perceived stigma and quality of life, based on a sample of TNBQ individuals residing in Italy. These findings carry important clinical implications: fostering patient–provider relationships that are perceived as affirming, respectful, and culturally competent may support TNBQ individuals in navigating and reducing barriers to healthcare access and utilization. In turn, this may help mitigate the harmful effects of perceived stigma, ultimately contributing to improved health and quality of life outcomes for TNBQ populations.

## Figures and Tables

**Table 1:** Participants’ sociodemographic and health-related characteristics.

*Gender*				
	Binary (*n* = 48)	Nonbinary (*n* = 84)	Total Sample (*n* = 132)	*p*
*Age (M* ± *SD)*	31 ± 11.1	27.1 ± 6.4	28.5 ± 8.6	<0.001
*Sex assigned at birth*				0.200
Male	14 (29.2%)	14 (16.7%)	28 (21.2%)	
Female	34 (70.8%)	68 (81%)	102 (77.3%)	
Intersex	–	2 (2.4%)	2 (1.5%)	
Total	48 (100%)	84 (100%)	132 (100%)	
*Educational level*				0.470
≤high school	22 (45.8%)	33 (39.3%)	55 (41.7%)	
≥college	26 (54.2%)	51 (60.7%)	77 (58.3%)	
Total	48 (100%)	84 (100%)	132 (100%)	
*Ethnicity*				0.727
Caucasian	47 (97.9%)	81 (96.4%)	128 (97%)	
Non-Caucasian	1 (2.1%) ^a^	3 (3.6%) ^b^	4 (3%)	
Total	48 (100%)	84 (100%)	132 (100%)	
*Chronic disease(s)*				0.209
Yes	10 (20.8%)	26 (31%)	36 (27.3%)	
No	38 (79.2%)	58 (69%)	96 (72.7%)	
Total	48 (100%)	84 (100%)	132 (100%)	
*Habitual source of primary care*				0.967
Yes	39 (81.3%)	68 (81%)	107 (81.1%)	
No	9 (18.8%)	16 (19%)	25 (18.9%)	
Total	48 (100%)	84 (100%)	132 (100%)	

*Notes.* Group differences in age were assessed through Student’s *t*-test. Group differences in all other variables were assessed through the *χ*^2^ test. Abbreviations: *M* = mean; *SD* = standard deviation. ^a^ = one Latine person; ^b^ = one Black, one Latine, and one unspecified ethnicity.

**Table 2:** Correlations and independent samples *t*-tests concerning perceived stigma, patient–provider relationship quality, barriers to care, and quality of life in binary and nonbinary groups.

					Binary	Nonbinary	*t*	*95% CI*	*Cohen’s d*
	1	2	3	4	*M* ± *SD*				
1. Perceived stigma	–				2.50 (0.70)	2.48 (0.69)	0.13	−0.23, 0.26	0.69
2. Patient–provider relationship quality	−0.35 ***	–			2.70 (0.67)	2.45 (0.57)	2.23 *	0.03, 0.46	0.61
3. Barriers to care	0.37 ***	−0.34 ***	–		2.02 (0.64)	1.96 (0.53)	0.62	−0.14, 0.27	0.57
4. Quality of life	−0.44 ***	0.27 **	−0.42 ***	–	0.79 (0.20)	0.70 (0.24)	2.09 *	0.00, 0.17	0.23

*Notes. M* = mean; *SD* = standard deviation; * = *p* < 0.05; ** = *p* < 0.01; *** = *p* < 0.001.

**Table 3:** Results of the serial mediation analysis subdivided by subpopulations.

	*β*	*SE*	*95% CI*	*p*
*Outcome: Patient–provider relationship quality*				
Perceived stigma				
Binary	−0.54	0.13	−0.80, −0.28	0.000
Nonbinary	−0.16	0.08	−0.33, 0.00	0.055
*Outcome: Barriers to care*				
Perceived stigma				
Binary	0.30	0.14	0.03, 0.57	0.034
Nonbinary	0.23	0.08	0.07, 0.40	0.006
Patient–provider relationship quality				
Binary	−0.29	0.14	−0.56, −0.02	0.038
Nonbinary	−0.18	0.11	−0.40, 0.03	0.096
*Outcome: Quality of life*				
Perceived stigma (direct effect)				
Binary	−0.11	0.04	−0.19, −0.02	0.015
Nonbinary	−0.12	0.04	−0.20, −0.05	0.000
Patient–provider relationship quality				
Binary	−0.03	0.04	−0.11, 0.06	0.504
Nonbinary	−0.00	0.05	−0.09, 0.09	0.947
Barriers to care				
Binary	−0.14	0.05	−0.23, −0.05	0.004
Nonbinary	−0.13	0.05	−0.22, −0.03	0.009
*Indirect effects*				
1. Perceived stigma → patient–provider relationship quality → quality of life				
Binary	0.05	0.09	−0.14, 0.24	
Nonbinary	0.00	0.02	−0.05, 0.05	
2. Perceived stigma → barriers to care → quality of life				
Binary	−0.14	0.09	−0.34, 0.00	
Nonbinary	−0.08	0.05	−0.20, −0.01	
3. Perceived stigma → patient–provider relationship quality → barriers to care → quality of life				
Binary	−0.08	0.04	−0.17, −0.00	
Nonbinary	−0.01	0.01	−0.03, 0.00	
*Total effect*				
Binary	−0.16	0.04	−0.08, −0.54	0.000
Nonbinary	−0.16	0.03	−0.23, −0.09	0.000

*Notes: β* = regression coefficient; *SE* = standard error; *CIs* = confidence intervals. The analysis was controlled for age, educational level, presence of chronic diseases, and habitual primary care access.

## Data Availability

Research data supporting the findings of the current study are available upon reasonable request from the corresponding author.
